# Non-invasive Bioluminescence Monitoring of Hepatocellular Carcinoma Therapy in an HCR Mouse Model

**DOI:** 10.3389/fonc.2019.00864

**Published:** 2019-09-11

**Authors:** Zhu Zhao, Juji Dai, Yan Yu, Qian Zhang, Sai Liu, Guanmeng Huang, Zheng Zhang, Tianke Chen, Rulu Pan, Liting Lu, Wenyi Zhang, Wanqin Liao, Xincheng Lu

**Affiliations:** School of Basic Medical Sciences, Wenzhou Medical University, Wenzhou, China

**Keywords:** hepatocellular carcinoma, animal model, hepatocarcinogenesis reporter mouse, bioluminescence imaging, drug efficacy evaluation

## Abstract

Animal models play crucial roles in the development of anticancer therapeutics. The ability to quickly assess the localized primary hepatocellular carcinoma (HCC) status in a non-invasive manner would significantly improve the effectiveness of anti-HCC therapeutic studies. However, to date, animal models with this advantage are extremely scarce. In this study, we developed a novel animal model for the fast assessment of drug efficacy against primary HCC *in vivo*. HCC was induced in immunocompetent hepatocarcinogenesis reporter (HCR) mice by diethylnitrosamine (DEN) injection and confirmed by histopathological staining. Using the bioluminescence imaging (BLI) technique, HCC progression was longitudinally visualized and monitored in a non-invasive way. Tests of two clinical drugs showed that both sorafenib and oxaliplatin significantly inhibited the BLI signal in mouse liver in a dose-dependent manner. The *in vivo* intensity of BLI signals was highly consistent with the final tumor burden status in mouse liver after drug treatment. The inhibitory effect of anti-HCC drugs was accurately evaluated through *in vivo* BLI intensity detection. Our study successfully established a bioluminescence mouse model for non-invasive real-time monitoring of HCC therapy, and this HCR mouse model would be a useful tool for potential anti-HCC drug screening and new therapeutic strategy development.

## Introduction

Hepatocellular carcinoma (HCC) is the most prevalent malignant cancer of the liver and ranks as the second leading cause of cancer-related death in the world (1, 2). Although the treatment and diagnosis of HCC have improved in the last two decades, the overall HCC patients' outcome is discouraging, with a 5-year survival rate of <20% (3, 4). The poor prognosis of HCC is attributed to its aggressiveness and tendency toward metastasis and recurrence, as well as the lack of effective therapeutics (4, 5). The multikinase inhibitor sorafenib is the only agent approved by the Food and Drug Administration (FDA) for advanced HCC in patients. However, the prognosis for patients remains dismal because the response rate to sorafenib is <5% (6–8). In recent years, although several clinical studies have found that nivolumab and regorafenib have beneficial effects on the survival of patients with advanced HCC, there is still no established effective drug to replace sorafenib (9–11). Hence, there is an urgent need to develop novel effective therapeutics for this disease (8).

Animal models are important tools for studying the pathogenesis, development, and therapeutic response of cancer (12–14). One of the best characterized, widely used animal models is the mouse model (15, 16). A number of liver cancer mouse models have been developed to mimic human HCC, each with advantages and disadvantages (17–19). Because it is simple to establish and the size of the tumor is relatively easy to monitor, the subcutaneous xenograft model is widely used in most experiments aiming to discover potential anti-tumor therapeutics. However, its major disadvantage is the lack of interaction between the tumors and liver tissue (19). Another limitation of the subcutaneous xenograft cancer model is that the nude mice used have impaired immune systems, so the model cannot represent the behavior of naturally occurring cancer in humans (20). This is of particular concern because the absence of an immune response and tumor–host relationship may lead to an abundance of false-positive responses in drug testing experiments (19). Orthotopic xenograft models, including carcinogen-induced or genetically engineered mouse models, are superior to the subcutaneous xenograft model in terms of replicating the tumor microenvironment (19). However, their major defect is that the HCC tumor volume cannot be measured directly unless the mice are sacrificed (15, 18, 19). Moreover, the high variability of liver cancer progression in these animal models demands large cohorts of animals to obtain data with adequate statistical significance (20, 21). Thus, the development of novel animal models that recapitulate the natural behavior of human HCC and its clinical response to therapy constitutes a major prerequisite for rapid anticancer drug screening and novel therapeutic strategy development (20).

Molecular imaging techniques (nuclear, fluorescence, and bioluminescence) are convenient biomedical tools that enable the visualization and quantification of biologic processes in a living organism (22, 23). Bioluminescence imaging (BLI) based on reporter gene expression can elucidate tumor-specific events or processes and thus has been applied extensively in cancer research (24, 25). The sensitivity of BLI, along with its ability to longitudinally monitor individual mice, makes it a useful tool for assessing tumor burden and therapy response (26). Presently, several BLI transgenic mouse models have been established to observe liver cancer *in vivo*, but none have been applied for anti-HCC drug development (27–29). In our previous study, we developed a hepatocarcinogenesis reporter (HCR) mouse model, which was established by placing a luciferase (luc) reporter gene cassette under transcriptional control of the endogenous Afp promoter (30). Diethylnitrosamine (DEN)-induced liver cancer was accurately detected using this luc-based BLI technology (30, 31). In the current study, we further refined the mouse model to longitudinally monitor HCC progression and developed a bioluminescence mouse model for non-invasive evaluation of the therapeutic effect of anti-HCC drugs.

## Materials and Methods

### Animal Maintenance and HCC Induction

All animal protocols were performed in accordance with the guidelines of the Institutional Animal Care and Use Committee of Wenzhou Medical University. HCR mice were maintained on a C3H genetic background. The male littermates were subjected to a single i.p. injection of DEN (20 mg/kg body weight, Sigma) 14 days after birth. The occurrence of HCC was identified by H&E staining 8 months after DEN injection, and HCC progression was monitored via BLI weekly for a total of 4–8 weeks.

### BLI

All BLI images were acquired using a Series Lumina II IVIS imaging system (PerkinElmer). BLI signals were collected 15 min after a single i.p. injection of D-luciferin (150 mg/kg body weight, Sinochrome) in PBS. During BLI signal collection, the mice were sedated via isoflurane gas inhalation. *Ex vivo* BLI of isolated livers was performed immediately after euthanasia of the animals.

### Drug Therapy

Oxaliplatin (OXA, S1224) and sorafenib (SOR, S7397) were obtained from Selleck and dispensed according to the instructions. Seven months after DEN injection, mice exhibiting a BLI intensity of 0.5–1.0 × 10^6^ photons/second/cm^2^/steradian (p/s/cm^2^/sr) were chosen and divided randomly into six groups (*n* = 8). According to a previous report (32), one set of mice received sorafenib at either 10 or 20 mg/kg or vehicle (volume of 5% PEG400+45% DMSO in water) once daily (*n* = 8 per treatment group). Treatments were given orally via gavage. A second set of mice received oxaliplatin at 5 or 10 mg/kg or vehicle (5% glucose in water) once a week (*n* = 8 per treatment group) as described in a previous study (33). Treatments were given by intraperitoneal injection. Animals were monitored weekly via BLI. At the end of the treatment and after the *in vivo* BLI intensity was recorded, the mice were sacrificed, and *ex vivo* BLI of the isolated livers was performed immediately after euthanasia of the animals. Individual liver tumor nodules were counted, and their size was measured. Tumor volumes were calculated as *V* = A^*^B^2*^0.5326 (*A* = long axis, *B* = short axis). The total tumor volumes are reported as the sum of all liver nodule volumes per mouse.

### Statistical Analysis

The data are presented as the mean ± SD, and statistical analyses were performed using one-way ANOVA for multiple group comparisons and *t*-test for two-group comparisons. Correlation analysis was performed using Pearson's correlation test. Differences with *P* < 0.05 were considered statistically significant.

## Results

**Figure 1:**
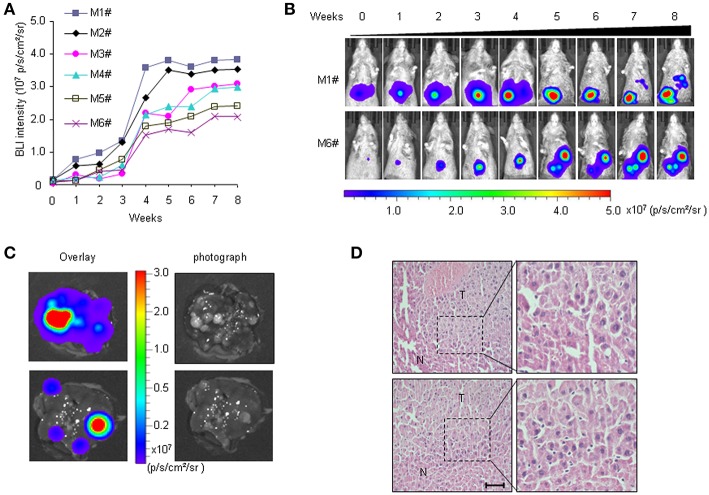
Non-invasive visualization of HCC progression in HCR mice via BLI. **(A)** *in vivo* quantified serial BLI intensity in each mouse for 8 weeks (M1# to M6# represent six mice monitored). **(B)** Serial bioluminescence imaging of liver cancer in representative HCR mice according to the time course [M1# and M6# represent the mice from **(A)**]. **(C)** *Ex vivo* BLI intensity and the presence of tumor nodules after 4 weeks of BLI monitoring. The photograph shows multiple visible liver tumors upon autopsy (right), most of which were luc-positive based on *ex vivo* BLI (left). **(D)** Histological analysis of liver samples from HCR mice after 4 weeks of BLI monitoring. Liver cancer nodules bearing strong BLI intensity (1.0–5.0 × 10^7^ p/s/cm^2^/sr) were stained with hematoxylin/eosin (H&E). Scale bar: 20 μm.

We previously established a bioluminescence HCR mouse model with luciferase gene expression restricted to liver cancer, which enabled BLI-mediated detection of hepatocarcinogenesis *in vivo* (30). In HCR mice, weak BLI signals began to appear 5 months after DEN injection. However, histological analyses revealed that most liver tumors induced by DEN were benign hepatomas until 6 months after DEN induction. In this study, more individual mice were included to observe longitudinal liver tumor progression with the aim of establishing an HCC therapy animal model. The primary data showed that at 7 months after DEN injection, medium-intensity BLI signals (0.5–1.0 × 10^6^ p/s/cm^2^/sr) were detectable in the majority of the HCR mice. To track HCC progression, we performed BLI to record the dynamic light intensity changes from this time point. The results showed that BLI signal intensity began to accumulate exponentially in each individual mouse during the first 4 weeks of detection. After that, the enhancement of BLI intensity rapidly attenuated. By the 7th week of detection, BLI signal intensity gradually stabilized and no longer increased (Figures 1A,B). The mice were executed at different time points to examine HCC progression. The results showed that 8 months after DEN induction (after 4 weeks of BLI detection), almost all mice with strong *in vivo* BLI signals (1.0–5.0 × 10^7^ p/s/cm^2^/sr) harbored a large number of tumor nodules in their liver. The *ex vivo* BLI data showed that the BLI activity was located exclusively in the tumor nodules of the liver, and livers exhibiting stronger BLI intensity harbored larger or more tumor nodules (Figure 1C). Histological analyses revealed that the majority of tumor nodules exhibiting strong BLI signals were malignant HCC (Figure 1D). Taken together, these data suggest that 7–8 months after DEN injection is an appropriate time period to observe the progression of HCC in HCR mice.

**Figure 2:**
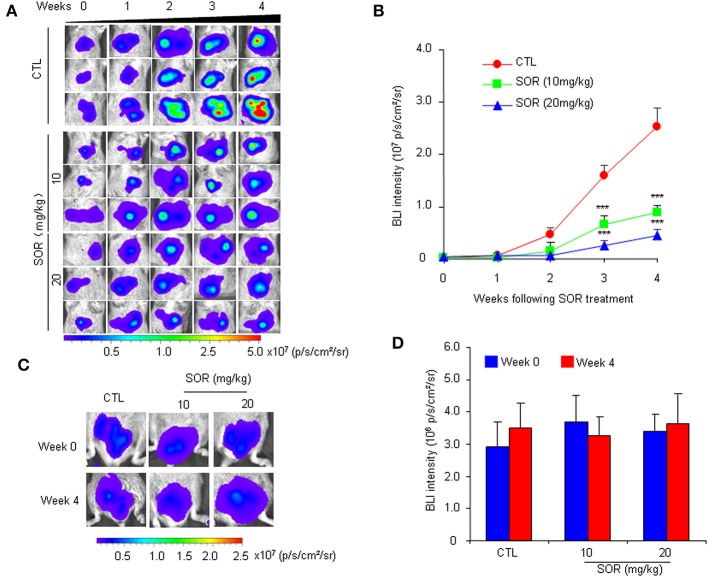
Non-invasive monitoring of BLI signals in the liver and testes area of HCR mice following sorafenib treatment.AQQ17 **(A)** Serial *in vivo* bioluminescence imaging of livers in representative mice treated with an oral dose of 10 or 20 mg/kg sorafenib (SOR) or vehicle control solution (CTL) for 4 weeks. **(B)** Quantified liver bioluminescence intensity in mouse groups that underwent vehicle or sorafenib treatment (*n* = 8). **(C)** The representative bioluminescence imaging of the testes area from mice treated with sorafenib (SOR) or vehicle solution (CTL). **(D)** Quantified bioluminescence intensity of the testes area from mice that underwent vehicle or sorafenib treatment for 4 weeks (*n* = 8). ***P < 0.001 vs. CTL.

**Figure 3:**
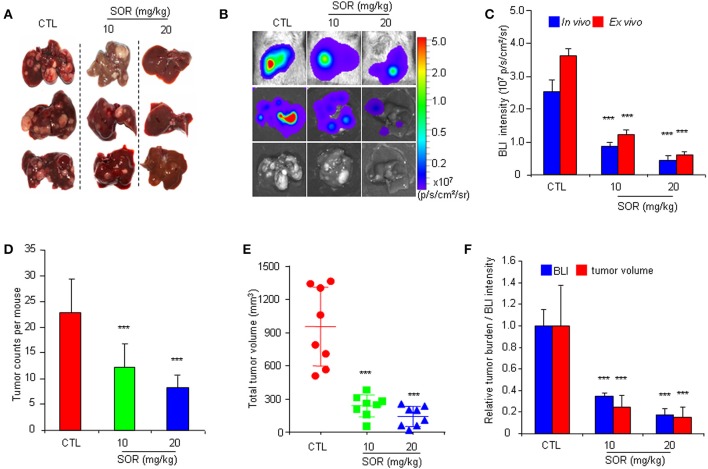
Comparative analysis of the effect of sorafenib on BLI intensity and the actual anti-HCC effects in HCR mice. **(A)** Gross appearances of representative livers with tumor nodules after 4 weeks of sorafenib (SOR) or vehicle solution (CTL) treatment. **(B)** Photograph showing the final *in vivo* and *ex vivo* BLI intensity and liver tumors upon autopsy. **(C)** The *in vivo* and *ex vivo* BLI intensity in mouse groups after sorafenib treatment (*n* = 8). **(D)** Group tumor burdens are presented based on the tumor count in each mouse (*n* = 8). **(E)** Group tumor burdens are presented based on the total tumor volume in individual mice (*n* = 8). **(F)** Relative tumor burden/BLI intensity in mice in groups that underwent vehicle (CTL) or sorafenib (SOR) treatment (*n* = 8). ***P < 0.001 vs. CTL.

To determine whether the HCR mouse model could be used to monitor the efficacy of anti-HCC drugs *in vivo*, we first conducted treatment experiments with sorafenib. Based on the above observations of HCC progression and BLI intensity changes in HCR mice, we chose mice with moderate *in vivo* BLI intensity (0.5–1.0 × 10^6^ p/s/cm^2^/sr) to begin the drug treatment experiment. Sorafenib was administered orally at a dosage of 10 or 20 mg/kg each day for 4 weeks, and vehicle solution was used as the control. During the 4-week test period, the *in vivo* BLI intensity in the control group increased rapidly, whereas the increase in the BLI signal was significantly attenuated in the sorafenib treatment groups starting at week 2 (Figures 2A,B). The most obvious difference was obtained at week 4, and the *in vivo* BLI intensity was dose-dependently inhibited in the sorafenib groups compared with that in the control group (Figure 2B). Interestingly, sorafenib had no significant effect on the BLI signal intensity that was consistently expressed in the adult male testis area (Figures 2C,D). These results suggest that the BLI intensity reduction should represent the therapeutic effect of sorafenib against liver cancer *in vivo*. To verify the feasibility of this speculation, we evaluated the inhibitory effect of sorafenib by quantifying the tumor burden in each liver based on tumor number and size and total tumor volume per mouse. The results showed that the *ex vivo* BLI signals and the number of tumors were apparently decreased in the sorafenib treatment groups (Figures 3A,B). The *in vivo* and *ex vivo* BLI signals were in agreement in each group (Figure 3C). In the sorafenib treatment groups, the tumor nodule count and total tumor volume were significantly reduced in a dose-dependent manner (Figures 3D,E). By comparing the BLI intensity and total tumor volume between the sorafenib treatment groups and the control group, we calculated the relative BLI intensity and tumor burden, respectively. The results demonstrated that the *in vivo* BLI intensity reductions were consistent with the inhibition rate of tumor burden (Figure 3F). Collectively, these data indicate that *in vivo* BLI signals can be applied to evaluate the therapeutic effect of sorafenib in HCR mice.

**Figure 4:**
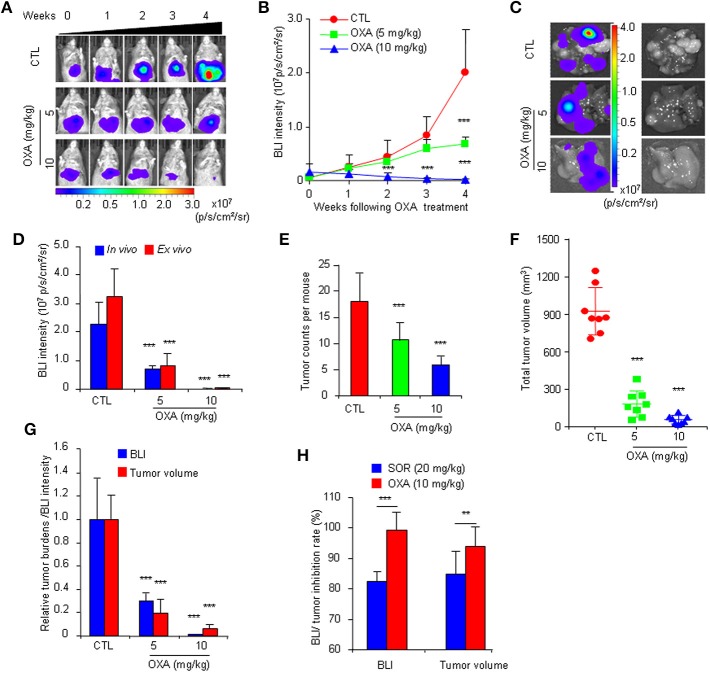
Non-invasive BLI monitoring of the tumor inhibitory effects of oxaliplatin in HCR mice. **(A)** Serial bioluminescence imaging of representative mice treated with 5 or 10 mg/kg oxaliplatin (OXA) or vehicle solution (CTL). **(B)** *in vivo* quantified bioluminescence intensity in mice that underwent vehicle or oxaliplatin treatment (*n* = 8). **(C)** Photograph showing the final *ex vivo* BLI intensity and liver tumors upon autopsy. **(D)** *In vivo* and *ex vivo* BLI intensity in mice after oxaliplatin treatment (*n* = 8). **(E)** Group tumor burdens are presented based on the tumor count in each mouse (*n* = 8). **(F)** Group tumor burdens are presented based on the total tumor volume in individual mice (*n* = 8). **(G)** Relative tumor burden/BLI intensity in mice that underwent vehicle or oxaliplatin treatment (*n* = 8). **(H)** BLI intensity/tumor inhibition rate of sorafenib and oxaliplatin (*n* = 8). **P < 0.01 vs. CTL, ***P < 0.001 vs. CTL.

**Figure 5:**
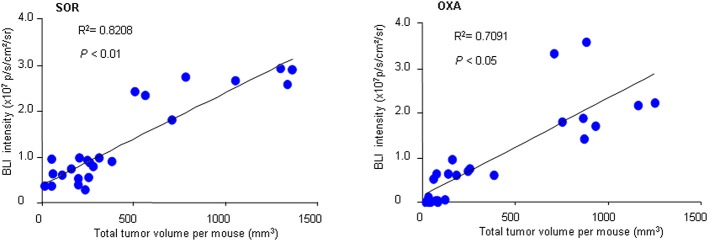
Correlation between BLI intensity and total tumor volume. At the end of sorafenib (SOR) or oxaliplatin (OXA) treatment, the *in vivo* BLI intensity and the total tumor volume for each mouse were calculated (*n* = 24). A correlation analysis between tumor volume and BLI intensity was performed using a Pearson correlation test.

To further confirm the practicality of this animal model, we conducted a similar experiment with another anticancer drug, oxaliplatin. HCR mice were administered oxaliplatin weekly via intraperitoneal injection. The results showed that oxaliplatin also significantly attenuated the increased *in vivo* BLI intensity and simultaneously suppressed liver tumor growth in a dose-dependent manner (Figures 4A–F), and the inhibitory effect of oxaliplatin on tumor burden was consistent with the *in vivo* BLI intensity decrease (Figure 4G). Interestingly, the 10 mg/kg oxaliplatin dose was more effective than the 20 mg/kg sorafenib dose in inhibiting tumor growth, and correspondingly, oxaliplatin had a stronger inhibitory effect on BLI signals than sorafenib (Figure 4H). These data suggest that the *in vivo* BLI signal can distinguish different efficacies of drugs. In addition, by analyzing the correlation between BLI intensity and tumor burden in each mouse, we found a direct positive correlation between BLI intensity and liver tumor burden in individual mice in both drug experiments (Figure 5). These results suggest that the reduction in BLI intensity could reliably reflect liver tumor burden status in individual mice following drug treatment. Overall, this study provides the proof of principle for using BLI imaging with HCR mice as a model for the non-invasive real-time assessment of anti-tumor drug efficacy in orthotopic HCC. This novel mouse model might be a useful tool for future anti-HCC therapeutic studies.

## Discussion

Animal models not only help to elucidate the pathophysiology of liver cancer but also can be employed for the development of new cancer drugs and therapeutic approaches (18). Because of their physiologic and genetic similarities to humans, their short lifespan, their breeding capacity, and the variety of manipulation methods available, mouse models are widely used for liver cancer research (15, 19). Multiple methods exist to induce liver tumor formation in mice, including genetically engineered mouse models, chemotoxic agents, intrahepatic, or intrasplenic injection of tumor cells, and xenograft approaches (34). However, no mouse model is ideal for all purposes. Each type of mouse model can only recapitulate hepatogenesis in some respects, leading to different apparent drug efficacies between preclinical drug testing and clinical application; the average rate of successful translation from rodent models to clinical cancer trials is <8% (20, 35).

We previously established an HCR mouse model using a genetic engineering technique and visualized early-stage liver cancer initiation and late-stage HCC existence via BLI (30). However, we found that most liver tumors induced by DEN at 6 months were benign hepatomas (30). Nine months after DEN induction, the mice were particularly susceptible to death from severe HCC tumor burden, and at the same time, the *in vivo* BLI signal was too strong to detect differences. In this study, we assessed HCR mice from 6 to 9 months after DEN induction to improve the technique to create a useful experimental model for simulating human HCC therapy. Our study found that 7 to 8 mouths is a suitable time period to simulate HCC progression and assess therapeutics in HCR mice. Starting from the 7th month, histopathological HCC was present in the majority of BLI-positive mouse livers. More interestingly, the BLI signal in individual mice increased sharply during the first 4 weeks of detection. After that, the enhancement of BLI intensity gradually ceased. These data suggest that these 4 weeks may represent a critical period of HCC progression. Therefore, we selected mice after 7 months of DEN induction to conduct a 4-week therapeutic experiment. Because bioluminescence is visible and can be quantified immediately, this allowed us to start the therapeutic experiment with mice bearing approximately identical and moderate luminescence intensity and avoided the disadvantages of variable HCC development onset in conventional mouse models.

In this study, we successfully developed HCR mice into a novel model for the non-invasive, real-time detection of anti-HCC drug efficacy. To demonstrate the conceptual feasibility of this animal model, we tested it with sorafenib and oxaliplatin, two first-line clinical drugs with known efficacy. In the experiments with both drugs, the expected results were observed. Sorafenib or oxaliplatin administration dose-dependently inhibited the *in vivo* BLI intensity in the liver. Moreover, the intensity of the BLI signal was highly consistent with the tumor burden in mice at the end of the experiments, and a direct positive correlation existed between BLI intensity and liver tumor burden in individual mice. These results suggest that the inhibitory effects of drugs can be directly evaluated by monitoring *in vivo* BLI intensity changes. In addition, as demonstrated in our previous report (30), BLI signals were consistently detected in the areas around the testes. Four weeks of sorafenib treatment had no significant effect on BLI intensity around the testis area. This result, in turn, indicates that the changes in BLI intensity in the liver are due to the inhibitory effect of sorafenib on tumors. Taken together, our study represents a successful attempt to track the therapeutic effects of drugs in live mice by following the growth of primary localized liver cancer using a non-invasive BLI technique. There are several important features of this approach. (1) The HCR mouse is a genetically engineered, immune-competent mouse model; thus, the DEN-induced primary HCC in this model is able to recapitulate many key biological features of human malignancy conditions (20, 36). (2) Simultaneous monitoring of localized liver cancer in a single mouse at multiple time points can provide accurate and reliable information. (3) The effectiveness of anti-tumor drugs can be assessed via BLI in a non-invasive manner. BLI provides a sensitive and convenient approach for non-invasively tracking tumor status in mice (37). This technique also enables us to monitor the tumor without sacrificing the animal and thus allows us to maximize data acquisition while using fewer animals than conventional approaches (21). (4) The longitudinal progression of HCC can be monitored in a real-time manner. Because of the stochastic and non-visible nature of HCC occurrence, conventional orthotopic models are often limited to showing only the end effect of anti-HCC drugs, rather than revealing the direct effect during the treatment process (38). Using the HCR mouse model, the BLI signals reflecting tumor behavior could be assessed throughout the course of the treatment. Due to the beneficial characteristics of real-time data collection, HCR mice enable the quick evaluation of a treatment and avoid unnecessary follow-ups. Finally, it is worth noting that our treatment period in this study represents mainly the early and middle stages of HCC progression, and further work is needed to determine whether it is effective for advanced HCC.

In summary, we demonstrated the application of a newly created HCR mouse model to evaluate the efficacy of anti-HCC drugs using a non-invasive real-time BLI technique. Primary HCC tumor behavior was rapidly and longitudinally visualized *in vivo*, and the efficacy of anti-HCC drugs was evaluated quickly by monitoring *in vivo* BLI intensity changes. This well-established HCR mouse model would likely be a useful tool for potential anti-HCC therapeutic screening, preclinical testing, treatment responsiveness monitoring, and novel therapeutic strategy development.

## Data Availability

The raw data supporting the conclusions of this manuscript will be made available by the authors, without undue reservation, to any qualified researcher.

## Ethics Statement

This study was carried out in accordance with the recommendations of the Institutional Animal Care and Use Committee of Wenzhou Medical University. The protocol was approved by the Institutional Animal Care and Use Committee of Wenzhou Medical University.

## Author Contributions

ZhuZ and JD performed the main experiments and analyzed the data. YY, QZ, SL, GH, ZheZ, and TC partially contributed to the animal experiments for this manuscript. RP and LL provided technical assistances. WZ and WL provided assistance in manuscript writing. XL conceived the study, designed the experiments, and wrote and finalized the manuscript.

### Conflict of Interest Statement

The authors declare that the research was conducted in the absence of any commercial or financial relationships that could be construed as a potential conflict of interest.

## Abbreviations

HCC: hepatocellular carcinoma
HCR: hepatocarcinogenesis reporter
BLI: bioluminescence imaging
DEN: diethylnitrosamine
FDA: Food and Drug Administration
Afp: alpha fetoprotein
luc: luciferase
p/s/cm^2^/sr: photons/second/cm^2^/steradian.
